# Mechanical Properties of 3-Hydroxybutyric Acid-Induced Vesicles

**DOI:** 10.3390/molecules28062742

**Published:** 2023-03-17

**Authors:** Seung Jun Jung, Kunn Hadinoto, Jin-Won Park

**Affiliations:** 1 Department of Chemical and Biomolecular Engineering, College of Energy and Biotechnology, Seoul National University of Science and Technology, Seoul 01811, Republic of Korea; 2 School of Chemical and Biomedical Engineering, Nanyang Technological University, Singapore 637551, Singapore

**Keywords:** 3-hydroxybutyric acid, mechanical properties, vesicles

## Abstract

The vesicle mechanical behaviors were studied upon its exposure to 3-hydroxybutyric acid using an atomic force microscope (AFM). Dipalmitoylphosphatidylcholine (DPPC) and 3-hydroxybutyric acid were used to manufacture the vesicles at their desired ratio. The deflection of an AFM probe with respect to its displacement was measured after characterizing the vesicle adsorption. The movement was analyzed with the Hertzian model to understand the physical behavior of the vesicles. However, in the deflection just prior to the first penetration, the model was a good fit, and the vesicle mechanical moduli were calculated. The moduli became lower with the higher ratio of 3-hydroxybutyric acid to DPPC, but the moduli were saturated at 0.5 of the ratio. These results appear to be the basis for the function of the metabolism associated with 3-hydroxybutyric acid, i.e., anesthetization and glycemic control, on the physical properties of cell membranes.

## 1. Introduction

3-hydroxybutyric acid (synonym, β-hydroxybutyric acid) is associated with diabetes and cranial nerve symptoms [1,2]. This compound is used as an alternative when the level of glucose is scarce in a body, because 3-hydroxybutyric acid is synthesized from acetyl-CoA in the liver [3]. The compound plays a role in inhibiting histone deacetylase and, thus, increases brain-derived neurotrophic factor (BDNF) [2]. This knowledge may be clinically relevant to the treatment of psychological symptoms. Furthermore, 3-hydroxybutyric acid has been reported to be an anesthetic in animals and has been reported to modulate the glycine-receptor function [4]. The anesthetic action was caused by the compound to modify mechanical behaviors of cell membranes to modulate the membrane protein function [5].

Since the phospholipids distributed in the biological membranes are associated with the vesicle-derived process and the antimicrobial action through their physical behavior, the mechanical properties are critical to the biological phenomena [6,7,8,9]. An atomic force microscope (AFM) can provide the properties of a surface with the motion of an AFM probe at the sub-nano scale [10,11,12]. Especially for a surface against the probe, the probe approach has been considered to estimate steric and electrostatic properties of the sample surface. In addition, its retreat has been used to find the adhesiveness of the surface. Many investigations have been performed with AFM force data combined with theories such as Johnson-Kendall-Roberts theory, Poisson-Boltzmann theory, particle mesh Ewald method, Derjaguin-Muller-Toporov theory, and coarse-grained model [13,14,15,16,17]. In this study, it was aimed to investigate the mechanical properties of 3-hydroxybutyric acid-induced vesicles with respect to their ratio of 3-hydroxybutyric acid to lipids because little is known about the 3-hydroxybutyric acid metabolic mechanism in cell membranes. This research is relevant to fundamental biological functions of cellular processes such as anesthetic action and BNDF release.

## 2. Results

### 2.1. Suface Morphology

**Figure 1:**
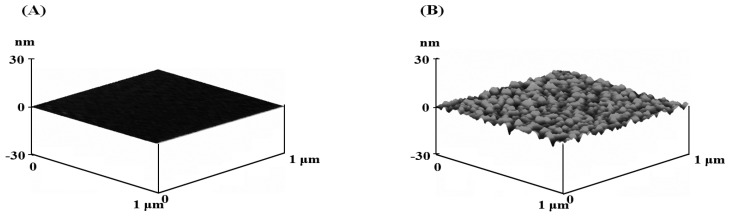
Surface morphology (**A**) before the lipid vesicle adsorption (**B**) after the adsorption.

The image prior to the adsorption suggested that the height change was less than 1 nm (Figure 1A), while the averages of the height and the width after the adsorption were 10 and 190 nm, respectively (Figure 1B). The lipid bilayer was around 5 nm thick, and the squeezed vesicles are made of two bilayers [18]. Therefore, the thickness of the adsorbed vesicles was around 10 nm. The flatness difference between two images indicated that the vesicles adsorbed to the mica surface. Furthermore, according to the ratio of 3-hydroxybutyric acid to DPPC, the morphology was little changed. Little change was predicted because the multi-layers were converted into the large unilamellar vesicle at 65 °C higher than the lipid transition temperature. The morphological characterization was consistent with the results of DLS that the diameter was little affected by the ratio of 3-hydroxybutyric acid to DPPC. Since the radius of the vesicle was included in the Equation (1), the effect of the ratio on the change in the morphology was considered.

### 2.2. Force Measurements

**Figure 2:**
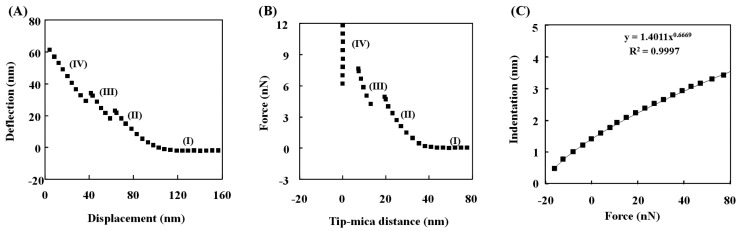
(**A**) Deflection with respect to displacement (z position) for the vesicle at 0% 3-hydroxybutyric acid; (**B**) force with respect to distance based on the data in (**A**); (**C**) indentation with respect to load force based on the region (II) data of (**A**,**B**).

The deflection data represented the physical phenomena of the vesicle. Figure 2A showed the deflection with respect to the displacement of the AFM probe and was transformed into the force versus the distance between the probe and the surface (Figure 2B). The force was acquired from the multiplication of the deflection by the probe spring constant, and the distance was determined from the subtraction of the deflection from the displacement.

These two graphs have identical distinct points which represent the sudden deflection of the probe and are divided into four different regions. In region (I), no contact of the probe occurred on the vesicle surface. Region (II) was from contact to the first vesicle penetration of the probe. Only region (II) was available to estimate the Young’s modulus of the vesicle. Region (III) was from the first penetration to the second vesicle penetration, and region (IV) was from the second vesicle penetration to contact on the surface. The ratio of the deflection to the displacement with the probe on a hard surface is theoretically about −1.0, and the ratio in the region (IV) was −0.99 [19]. Since the sterically repulsive distance was approximately 5 nm, no vesicle adsorption occurred on the probe. The ratio and the distance were mutually consistent with each other. In other words, the probe, after two penetrations, contacted the mica surface. This analysis was also identical to the results repeated in pure water.

### 2.3. Theoretical Analysis

The elasticity was explained with the slope of the force to the distance [16,20]. The slope corresponded to the resistance of each layer to the probe load. The slopes of region (II) and region (III) in Figure 2B are listed in Table 1 for the ratio of 3-hydroxybutyric acid to DPPC, and their variation ranges are less than 3%. The slope for the 0% 3-hydroxybutyric acid was 0.7 for region (II), while it was 3.6 N/m for region (III). The exponential value (b) was 0.667 for region (II) from the fitting δ = AF^b^ to the data, and it was 0.908 for region (III). In this formula, δ is the indentation [m] of the probe into the vesicle; F is the load force [N] of the probe; and A is the proportional constant from several parameters shown below. Therefore, the justification of the elasticity was performed using the values of b.

## 3. Discussion

Since the exponential value of the load force for the indentation was 0.667, it was confirmed that the physical behavior of the vesicle prior to the first vesicle penetration was elastic. This confirmation led to further analysis to estimate Young’s modulus, and the fits of the Hertzian model to data were the indentation (δ) for the load force (F) (Figure 2C). It was found for the physical behavior of the vesicle prior to the first vesicle penetration that the relation of the indentation to the force was in the range of exponential 0.656 to 0.676. Therefore, at least region (II) remained consistently elastic. From Young’s modulus, the bending modulus was estimated. The Equations (1) and (2) provided the two vesicle moduli of the vesicle.

The increase in the ratio of 3-hydroxybutyric acid to lipid led to the decrease in Young’s modulus until the ratio was 0.5. No further decrease was observed beyond 0.5. The vesicle without 3-hydroxybutyric acid showed 81 × 10^6^ Pa as Young’s modulus, which was consistent with the previous research [21]. Therefore, the dependence on the ratio was gradually decreased and saturated at 0.5. Interference of 3-hydroxybutyric acid in the lipid–headgroup arrangement may occur. The more 3-hydroxybutyric acid there is, the less moduli (Table 2). The value for each condition in Table 2 suggested an average along with a range of results.

The previous study showed that a greater portion of 3-hydroxybutyric acid induced higher fluid [5]. In that research, the transition from solid to liquid happened in the solid phase. Those results are identical with those of this research because the vesicle state was in a solid phase. In addition, it has been also suggested that the association with 3-hydroxybutyric acid generated the distribution effect on the membrane to cause the change in the lipid layer.

## 4. Materials and Methods

Dipalmitoylphosphatidylcholine (DPPC) and 3-hydroxybutyric acid were from Sigma Aldrich (St. Louis, MO, USA). The molecules of DPPC formed the multi-layers on a glass vial bottom by evaporating chloroform with nitrogen, in which they were dissolved. The addition of the aqueous solution of Hepes 2 mM and 3-hydroxybutyric acid at pH 7 was performed in the vial to cover the layers completely overnight. The solution was vortexed 4 times every 20 min at 60° and sent through a 100-nanometer pore membrane to acquire the vesicle. The solution through the membrane was transferred for the measurement of the vesicle diameter, which was between 130 and 170 nm.

An AFM probe was located in a liquid cell and approached the mica peeled and transferred previously to the top of the AFM scanner (Nanoscope v5.12, Bruker, Billerica, MA, USA). Prior to the approach, a silicon O-ring was placed on the mica. After the approach, the vesicle solution was added to cover the mica surface completely at room temperature. After 2 hours for the adsorption, the non-adsorbed vesicles were washed out by injecting the buffer solution into the inside. After characterizing the adsorption, the deflection data were collected between the probe and the vesicle at room temperature [22]. The following theory was considered for the data with twice clear penetrations only.

The selection of data was for the analysis with the Hertzian model, which describes the elasticity of the sphere with the equation below [20,23].

(1) $$\delta =0.825{\left[\frac{{\left(1-\nu_{\mathrm{ves}}^{2}\right)}^{2}(R_{\mathrm{tip}}+R_{\mathrm{ves}})}{E_{\mathrm{ves}}^{2}R_{\mathrm{tip}}R_{\mathrm{ves}}}\right]}^{\frac{1}{3}}F^{\frac{2}{3}}\;\delta =\left|z-z_{0}\right|-\left(d-d_{0}\right),\;F=k(d-d_{0})$$

where *ν*_ves_ is Poisson’s ratio of the vesicle; *R*_tip_ and *R*_ves_ are respectively the radius [m] of the probe and vesicle; *E*_ves_ is the Young’s modulus [Pa] of the vesicle; and *k* is the spring constant [N/m] of the probe. The indentation was acquired from the difference between the probe displacement |*z* − *z*_0_| [m] and the probe deflection (*d* − *d*_0_) [m]. *z*_0_ was the distance from the boundary between the regions, and *d*_0_ was the deflection of the boundary. Therefore, the fitting of the experimental data was used through the least-square method to calculate *E*_ves_ using Equation (1), which was applied to the equation below to estimate the bending modulus *k*_c_ [J].

(2) $$k_{c}=\frac{E_{\mathrm{ves}}h^{3}}{12(1-\nu_{\mathrm{ves}}^{2})}$$

where *h* is the thickness [m] of the vesicle bilayer.

## 5. Conclusions

The mechanical moduli of DPPC vesicles were investigated for the 3-hydroxybutyric acid ratio. The probe deflection was interpreted with the Hertzian model to investigate the physical behaviors of the DPPC vesicle neighboring 3-hydroxybutyric acid. The mechanical moduli were saturated at the 3-hydroxybutyric acid ratio of 0.5. This result may be caused by the degree of the lipids associated with 3-hydroxybutyric acid at the ratio. This study may be basis for biological mechanisms related to cellular processes such as anesthetization, glycemic regulation, and neuron responses.

## Figures and Tables

**Table 1:** Slopes of the force to the distance for the ratio of 3-hydroxybutyric acid to lipid.

Ratio of 3-Hydroxybutyric-Acid to Lipid	0	0.1	0.3	0.5	0.7	1.0
Slope of 1st steric force (N/m)	0.7 ± 0.01	0.69 ± 0.01	0.67 ± 0.01	0.65 ± 0.01	0. 65 ± 0.01	0.65 ± 0.01
Slope of 2nd steric force (N/m)	3.6 ± 0.01	3.5 ± 0.01	3.4 ± 0.01	3.3 ± 0.01	3.3 ± 0.01	3.3 ± 0.01

**Table 2:** Mechanical moduli of the vesicle for the ratio of 3-hydroxybutyric acid/lipid, *E_ves_*: Young’s modulus and *k_c_*: bending modulus.

	Ratio of 3-Hydroxybutyric Acid/Lipid					
	0	0.1	0.3	0.5	0.7	1.0
*E_ves_* × 10^6^ (Pa)	81 ± 2	80 ± 2	78 ± 2	76 ± 2	76 ± 2	76 ± 2
*k_c_* × 10^−19^ (J)	11.3 ± 0.3	11.1 ± 0.3	10.7 ± 0.3	10.5 ± 0.3	10.5 ± 0.3	10.5 ± 0.3

## Data Availability

Not applicable.
